# Should Ultrasound-Guided High Frequency Focused Ultrasound Be Considered as an Alternative Non-Surgical Treatment of Uterine Fibroids in Non-Asiatic Countries? An Opinion Paper

**DOI:** 10.3390/jcm11030839

**Published:** 2022-02-05

**Authors:** Luz Angela Torres-de la Roche, Sarah Rafiq, Rajesh Devassy, Hugo Christian Verhoeven, Sven Becker, Rudy Leon De Wilde

**Affiliations:** 1 University Hospital for Gynecology, Pius Hospital, University Medicine Oldenburg, 26121 Oldenburg, Germany; luz.angela.torres-de.la.roche@uol.de (L.A.T.-d.l.R.); drsarahrafiq@gmail.com (S.R.); 2 Centre of Excellence in Gynecological Minimal Access Surgery and Oncology, Dubai London Clinic and Speciality Hospital, Dubai 3371500, United Arab Emirates; rajeshdevassy@gmail.com; 3 Center for Endocrinology, Preventive Medicine, Reproductive Medicine and Gynecology, 40211 Düsseldorf, Germany; h.c.verhoeven@t-online.de; 4 University Hospital for Gynecology and Obstetrics, University Hospital Frankfurt, 60590 Frankfurt, Germany; sven.becker@kgu.de

**Keywords:** high intensity focused ultrasound (HIFU), uterine fibroids, focused ultrasound, uterine-sparing treatment, ultrasound guided intervention procedures

## Abstract

Minimally invasive interventions for myomata treatment have gained acceptance due to the possibility of preserving fertility with reduced trauma induced by laparotomy as way of entrance. There are insufficient data regarding outcomes of high intensity focused ultrasound (HIFU) in non-Asiatic women. Therefore, we revised the available evidence to present an expert opinion that could support physicians, patients and policy-makers for considering this approach in other populations. We revisited systematic reviews, randomized controlled trials and cohort studies from January 2018 to August 2021 using PubMed and Google scholar, regarding short and long term outcomes after ablation with focused ultrasound waves. In total, 33 studies, including 114,810 adult patients showed that outcomes of this approach depend on several parameters directly related with resistance to thermal ablation, especially fibroid size and vascularization. Two studies report satisfactory outcomes in Afro-American women. In accordance to the technique used, fibroid volume reduction showed to be higher in fibroids <300 cm^3^ after ultrasound guided HIFU than after MRI guided. Compared to myomectomy and uterine artery embolization, HIFU seems to have shorter hospital stay, higher pregnancy rates and similar adverse events rates, with skin burn being the most reported. Symptoms and quality of life improvement is similar to myomectomy but lower than embolization, however reintervention rate is higher after HIFU. Lacks evidence about long-term sarcoma risk after ablation. Available evidence shows that HIFU can be considered as a uterine sparing treatment for women of different ethnicities suffering of uterine myomatosis, especially for those wishing to preserve their fertility.

## 1. Introduction

In clinical practice, non-invasive gynecological interventions have gained acceptance due to the possibility of preserving fertility and reducing the risks associated with invasive surgery but with similar results with respect to symptoms resolution [1]. Recently, thermal ablation of tissues has been introduced to treat benign and malignant tumors by using different types of energies including radiofrequency current, micro-laser and ultrasound waves [2]. The latest, have been used based on their ability to penetrate deep into tissues and to induce molecular reactions through mechanical and thermal mechanisms [2]. The beam of high intensity focused ultrasound (HIFU) can be guided by MRI (MRgHIFU) or ultrasound (USgHIFU) to accurately localize and treat the lesion [3]. MRgHIFU has limited clinical application because the need of a radiologist at all times of the procedure and being more time consuming than USgHIFU [4]. Before starting therapy and to avoid skin burn and damage to nearby organs or tissues, an acoustic window is created, then the device brings the acoustic energy through the skin via a coupling gel or a water balloon [5]. The waves pass through the tissues and arrive at the target lesion, raising the temperature of the focal point leading to irreversible coagulative necrosis by absorption of ultrasound energy (thermal effect) and thereby ultrasound-induced cavitation damage (mechanical effect) [5]. The blood perfusion of the lesion is reduced and a defined border between the treated area and surrounding tissue is created [2]. These changes are easy to recognize post-procedure by contrast–enhance MRI or ultrasound. Moreover, membrane hormone receptors are impaired making them less sensitive to hormones, which in turn could prevent fibroids [6]. Though several cohort studies report HIFU outcomes to be better than surgery, the use of this technology is not worldwide approved, available or used [7].

In gynecology, HIFU is proposed for treating symptomatic uterine myomata [2,7,8], a benign disease that affects 33–77% of women worldwide, threatening their fertility and quality of life [9]. Furthermore, HIFU is being increasingly used in Asiatic countries as a non-invasive intervention for adenomyosis, and different primary malignant and metastatic tumors, such as hepatocellular carcinoma, breast, prostate and pancreatic cancer, small size renal tumors, carcinoma of esophagus, glioblastoma and bone tumors [2]. It is also considered as a treatment for non-cancerous lesions such as prostate hypertrophy, solid and complex thyroid nodules, blood–brain barrier disruption, intracerebral hemorrhage, glaucoma, atrial fibrillation and methicillin-resistant staphylococcus aureus-induced abscess [2]. Additionally, it is being investigated as an alternative treatment of various brain disorders such as Parkinson’s disease, essential tremor, Alzheimer’s disease, depression, anxiety and pain syndromes [2]. Undeniably, the scientific community should be aware of the possible advantages and risks of this novel technology.

Considering that gynecologists and patients are interested in having access to non-invasive alternatives for the management of uterine myomatosis, but having insufficient data on HIFU long-term effects, we present an evidence-based expert opinion that could support physicians, patients and policy-makers for eventually considering the HIFU as an alternative to surgery and uterine artery embolization (UAE) to treat patients presenting with uterine myomatosis. Evidence comparing HIFU with available medical therapies is out of the scope of this paper.

## 2. Materials and Methods

A search was conducted using the PubMed and Google scholar databases from January 2018 to August 2021. Systematic reviews, randomized controlled clinical trials and observational studies regarding outcomes of HIFU were collected. Outcomes of interest were: role of different technical parameters on symptoms resolution, adverse events, fibroid shrinkage, reintervention, pregnancy and risk of sarcoma. The following inclusion criteria were used: studies including women diagnosed with uterine fibroids (systemic review, meta-anmeta-analysis, randomized controlled trial, cohort studies, both comparative and non-comparative), published in English. Commentaries, case reports, technical reports, animal studies, letters to editor, published papers without an available full-text and non-English articles were excluded. Data Extraction and Outcome Measure

We selected articles according to the above mentioned inclusion and exclusion criteria. We scrutinized titles and abstracts and then gathered all potentially relevant studies for full text evaluation. We extracted information about authors, year of publication, study design, interventions, participant age, participant ethnicity, post-procedure results. To illustrate the safety and efficacy of HIFU, we considered outcome measures such as, complications, adverse events, serious adverse events, time to recovery, hospital stay, time to tumor remission, pregnancy rate, reintervention rate and sarcoma-risk.

## 3. Results

**Figure 1:**
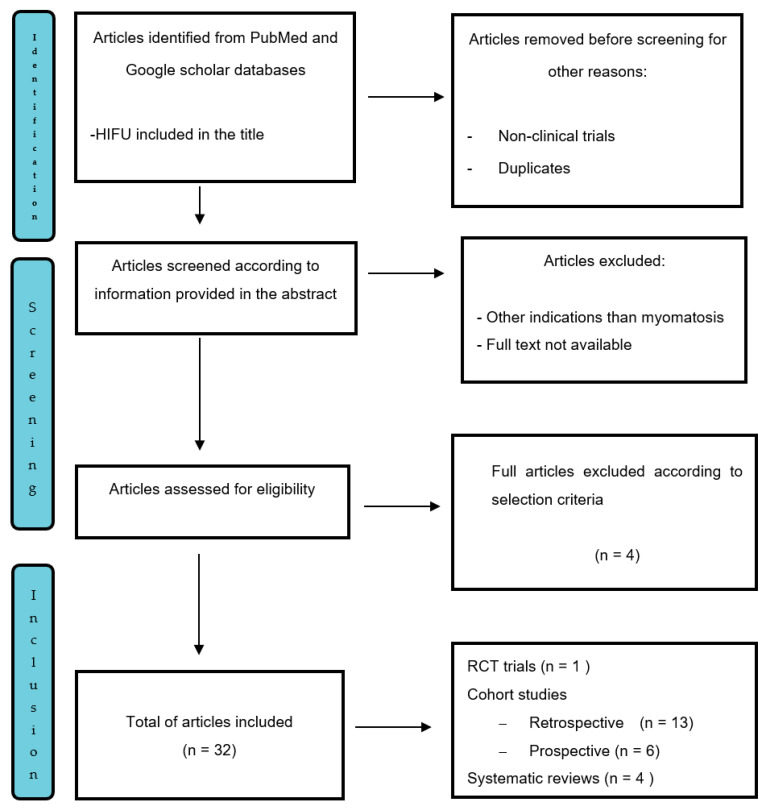
Prisma flow diagram showing the selection of articles regarding high frequency focused ultrasound (HIFU) for uterine fibroid treatment. Patient Characteristics.

In total, 47 studies were retrieved and identified for possible evaluation (Figure 1). Duplicates and studies that not met the inclusion criteria were removed. A total of 32 met the inclusion criteria and remained for full text reviews. Overall, four of these were systemic reviews, six were meta-analysis, eight were prospective clinical trials and 14 were retrospective cohort studies. In addition, seven articles describing technical parameters that affect HIFU outcomes were analyzed.

A total of 32 studies representing 114,810 women with symptomatic uterine fibroids underwent HIFU were revisited (Table 1 and Table 2). In total, 19 were conducted in China, three in The Netherlands, two in Taiwan, one in Hong Kong, two in USA, two in Vietnam, one in Finland, two in South Africa, one in Canada. Most of the studies evaluate treatment outcomes on the basis of resolution of symptoms, complications, fibroid volume reduction, reintervention rate for recurrent symptoms and relapse of disease, and pregnancy outcomes. All studies considered patients aged more than 18 years (range 26 to 51 years).

### 3.1. Ethnicity

Most of the reported outcomes are from studies conducted in Asiatic populations. Only two studies had specifically reported the ethnicity of their participants. Zhang CHJ, et al. [4] evaluated efficacy and safety of HIFU in 26 premenopausal African women. The study showed a 52.5 ± 36.3% tumor reduction in six months, no major complications and two reinterventions due to persistent heavy menstrual bleeding [4]. Suomi V, et al. [10] reported treatment outcomes in Asian, White and Black population. However, in this study most of the patients were white (80/89) and the results were not adjusted according to ethnicity [10].

### 3.2. Non-Perfused Volume Ratio

Non-perfused volume ratio (NPVR) is defined as percentage of non-perfused volume (NPV) in the uterine fibroids on MRI done after USgHIFU or MRgHIFU; that is, NPV divided by original tumor volume targeted. It is considered as a marker for assessing the efficacy and primary outcome of HIFU ablation [11,12]. Fan HJ et al. [11] reported that the goal of HIFU treatment should be to get maximum NPVR which is measured immediately through MRI by calculating fibroid shrinkage volume (FVS) after procedure and at the 1st and 3rd month on follow-up visits. They observed a NPVR variation between 16.7 to 97.9% (SD 74.7 ± 15.1%) in 207 patients. Higher NPVR was achieved by patients having fibroids in anterior location, hypointensity SI on T2WI and anteverted uterine position. Transmural myomata, hyperintensity SI on T2WI, longer distance from myoma’s ventral side to skin and posterior fibroids were more difficult to ablate and showed low NPVR [11].

### 3.3. Thickness of Abdominal Subcutaneous Tissues

Four studies demonstrated that the subcutaneous fat layer attenuates ultrasound energy [5,36]. Heating efficacy of ultrasound field is significantly reduced as fat tissues easily absorb thermal energy, which potentially causes thermal injury and low NPV ratio [5,36]. Additionally, total treatment time become longer as a thick fat layer affects cooling time between sonication (abdominal wall thickness: OR = 1.570, 95% CI= 1.329–1.854; *p* = 0.000 and BMI OR = 2.097, 95% CI= 1.575–2.745; *p* = 0.000) [5]. Abdominal wall thickness, BMI, sonication time per hour (time of ablation when energy was being delivered to the target) and total energy applied are potential factors for thermal damage in abdominal wall structures [13]. Moreover, thickness of abdominal subcutaneous tissues was second in the ranking feature selection method proposed by Suomi V, et al. [10], better than patient weight (rank 13) and height (rank 15), despite subcutaneous fat thickness being strongly correlated with weight, as total weight includes fat and muscle mass and does not reflect localized fat content.

### 3.4. Perfusion of Fibroids

Ablation capability of HIFU depends on perfusion characteristics of fibroids like blood flow velocity and perfusion volume, which could be evaluated before procedure by contrast enhanced ultrasound (CEUS) [30]. More perfusion to fibroids means more resistance towards HIFU [12]. Wang YJ, et al. [12] reported that myomata which showed lower blood flow velocity and higher perfusion volume have fast rise and decline in blood flow, and therefore were more resistant to HIFU and had a reduced ablation effect.

### 3.5. Energy Efficiency Factor

The energy efficiency factor (EEF) represents the ultrasonic energy required for uterine fibroids ablation per unit volume (1 mm^3^) [11]. It reflects the energy deposition efficiency of HIFU and is considered one of the most accurate quantitative indicators of HIFU ablation. The smaller the EEF, the lower energy needed to ablate a volume of uterine fibroids and to achieve a higher HIFU ablation efficiency [11]. Different factors are negatively correlated to EEF, including: size of uterine fibroids, anterior location of myoma, hypointensity SI on T2WI, positive co-relation with distance from the uterine fibroid ventral side to skin, enhancement type on T1WI and transmural type of myomata [11]. It is more difficult to ablate myomata with long distance from its ventral side to skin, in retroverted uterus, significant enhancement on T1WI, hyperintensity SI on T2WI, small size, transmural type and posterior location [11], and, in the same way, correlated with NPVR.

### 3.6. Resolution of Symptoms, Quality of Life and Hospital Stay

Several studies evaluated efficacy by using a validated health and symptom related quality of life questionnaire (UFS-QoL) [3,8,14,15,16,17,18,37] or transformed symptom severity scale (tSSS) [15,19], applied before and up to six months after HIFU (Table 3).

Wang Y et al. [15] observed a higher symptomatic relief in the USgHIFU than in myomectomy group (95.9% vs. 89.1%), with a mean decrease of 30.5 points at the tSSS scale from baseline 40 (range 12–66) to 10.2 (range 0–14) during six months follow-up as well as, a lower symptom recurrence rate (11.9% vs. 27.8%) [15]. Similarly, Chen J, et al. [37] showed that QoL was improved significantly in the HIFU group in three aspects, bodily pain, vitality and emotions, as compared to myomectomy, at six months (10.20 ± 10.18 vs. 7.09 ± 8.25; *p* = 0.000) and 12 months (7.73 ± 9.65 vs. 5.77 ± 7.77). Thereafter all groups showed no differences in QoL. In the non-comparative study from Lozinski T et al., 2021 [19] a 69% improvement in the uterine fibroid-related symptoms scale was observed at third month post MRgHIFU, which increased up to 76% at sixth month, which were directly related with fibroid size reduction. Similar results were found in the meta-analysis from Sandbreg EM, et al. [31] and Verpalena IM et al. [32].

In comparison to UAE, the meta-analysis conducted by Liu L, et al. [8] found that the effect of UAE was superior in terms of alleviation of myoma-related symptoms and improvement in QoL for treatment with a significantly decrease in symptomatology and increase in QoL in UAE group (overall mean difference 19.54; 95% CI 15.21–23.13; *p* < 0.001; I^2^ = 73%). However, patients were older in UAE group (41.2 y-o; range 33.6–55.3 vs. HIFU 36.1 y-o; range: 27.7–41), which affects the observed difference.

Regarding hospital stay, Chen J, et al. [37] reported that HIFU showed better results as compared to surgical intervention, with faster return to work and lower cost.

**Table 3:** Uterine fibroid size reduction and symptomatic relief after HIFU and other uterine sparing intervention.

Author [Ref]	UFS before Therapy	UFS after Therapy	*p*-Value	QoL Score before Therapy	QoL Score after Therapy	*p*-Value
Jeng CJ et al., 2020 [3]	63.9 ± 29.9	45.3 ± 26.9	0.000	NA		
Chen J et al., 2018 [37]	HIFU: 19.89 ± 14.29 LM: 15.34 ± 13.34	**6 months**: HIFU: 10.20 ± 10.18 LM: 7.09 ± 8.25	0.000	**Baseline** HIFU: 72.75 ± 16.33 LM: 72.85 ± 14.46	**6 months**: HIFU 82.48 ± 12.94 LM: 80.44 ± 12.41	0.000
		**12 months**: HIFU: 7.73 ± 9.65, MYO: 5.77 ± 7.77	NA		**12 months**: HIFU: 85.84 ± 12.22 LM: 83.45 ± 11.28	0.000
Cheung VYT et al., 2019 [14]	USgHIFU: 27 (21–33)	3 months: 16.5 (11–23)	0.0001	NA		
		6 months: 16 (8–21)	0.0002			
		12 months: 14.5 (8–30)	0.0002			
Wang Y et al., 2020 [15]	tSSS: 34 (10–60)	tSSS: 40 (12–66)	0.178			
Laughlin-Tommaso SL et al., 2020 [16]	MRgHIFU: 53.9 (19.8)	**6 months** MRgHIFU: 31.3 (18.7)	<0.001	77.0 QoL score		<0.001
	UAE: 53.1(19.8)	UAE:13.2 (10.2)		91.2 QoL score		
		**12 months**: MRgHIFU:34.1 (24.7)	<0.001	72.8 QoL score		<0.001
		UAE:13.8 (12.8)		93.0 QoL score		
He M et al., 2018 [17]	56.3 ± 16.7	1 months: 40.5 ± 17.2	NA	41.3 ± 21.2	1 month: 60.6 ± 19.7	NA
		3 months: 31.0 ± 15.1	NA		3 months: 72.3 ± 18.1	
		6 months: 20.6 ± 14.2	NA		6 months: 73.4 ± 19.2	
Lee JY et al., 2019, JY [18]	USgHIFU 93.1 ± 32.5	72.6 ± 26.6	0.0001			
Verpalena IM et al., 2019 [32]	MRgHIFU 46.1 (33.7–58.4)	NA		6-months: 56.1 (50.0–62.2)		NA
		NA		12-months: 53.6 (41.8–65.5)		NA
Lozinski T et al., 2021 [19]	NA	3 months: NA 6 months: 87.44 ± 1.96%	NA	NA	**3 months**: Better 61% Much better: 8% **6 months**: Better: 53% Much better: 23%	NA

HIFU: high intensity focused ultrasound; MRgHIFU: MRI guided HIFU; USgHIFU: ultrasound guided HIFU; LM: laparoscopic myomectomy; UAE: uterine artery embolization; UFS: uterine fibroid score; QoL: quality of life; tSSS: transformed symptom severity scale; NA: not available.

### 3.7. Adverse Events

Several studies report that complication rates after HIFU are low (<1%) and decline over time, from 0.9565% to 0.2852% over seven years [3,22,28,37], which is attributed to the learning curve of physicians [21]. A list of the complications and short-term adverse events (AE) reported from different studies are shown in Table 4 and Table 5.

Major complications are infrequent or rare, being skin burn, fever and venous thrombosis the most reported, which usually resolve without sequela. Bowel injuries can occur when the bowel is in the acoustic pathway or when fibroids are over treated, being diagnosed up to 12 days after therapy [20,21]. Pubic symphysis pain and hydronephrosis are reported to appear after treating cervical fibroids, due to transient increase of fibroid size and edematous compression of ureter [21]. In addition, peritoneal adhesions after HIFU, have been reported to develop in the same frequency, location and severity than after surgical myomata removal (43.75% vs. 36.14%; *p* = 0.132) [20].

In comparison to other conservative therapies, Jeng CJ, et al. and Liu L, et al. found no significant differences in HIFU and myomectomy (pooled OR: 0.11, 95% CI: 0.00–4.41, *p* < 0.01) [3], or with UAE [8] (overall RR 3.42; 95% CI, 0.07–158.04; I^2^ = 86%; UAE 40.0% and HIFU 46.5%; *p* = 0.53), respectively. Specifically, for HIFU less AE were observed than in UAE patients (OR = 0.736; 95% CI, 0.203–2.670, *p* = 0.641) [22].

Minor complications are attributed to the inflammation response to the thermal effect of HIFU, including mild lower abdominal pain, sacrococcygeal pain and abnormal vaginal discharge, subsiding in few days, as reported by several studies (Table 5) [3,4,13,14,17,22,32,37]. Some factors not related with the thermal effect could lead to interruption of the MRgHIFU, including stress, impatience or claustrophobia [20]. Discharged necrotic tissue usually began in the first cycle after therapy and resolve spontaneously within eight months after therapy [13,17,31], which was observed in up to 60% of patients. The meta-analysis from Jeng CJ et al. [28] reports that abdominal pain and abnormal vaginal discharge are more prevalent in MRgHIFU (mean 37.0% and 20.3%, respectively) as compared to USgHIFU (mean 31.2% and 11.3%, respectively), as timely adjustments could be made according to patient’s pain feedback during USgHIFU. In contrast, sciatic nerve pain and thermal skin injury was found to be less frequent in MRgHIFU, according to the meta-analysis of Yu L et al. [29].

**Table 5:** Frequency of minor adverse events after HIFU therapy reported in clinical studies.

Author [Ref]	Minor Adverse Event	(%)
Jeng CJ et al., 2020 [3]	Vaginal secretion	0.086
	Low abdominal pain	0.022
	Leg and buttock pain	0.0072
	Hematuria	0.0053
	Uterine bleeding	0.0022
	Blurred vision	0.0001
He M et al., 2017 [17]	Transient leg pain	0.13
	Buttock pain	0.43
	Skin burning sensation	0.61
	Lower abdominal pain	0.60
	Abnormal vaginal discharge	0.6
Cheung VYT et al., 2019 [14]	Second degree skin burn	0.05
	Urinary tract infection	0.05
	Pelvic and back pain	0.35
	Intense lower abdominal pain	0.05
Yin N et al., 2018 [13]	Lower abdominal pain	0.38
	Buttock pain	0.19
	Vaginal bleeding and discharge	0.16
	Lower limb paresthesia	0.048
	Urinary retention	0.003
	Fever	0.006
	Hematuria	0.0056
Chen J et al., 2017 [37]	Lumbar and back pain	0.011
	Numbness and pain in lower limb	0.025
	Weakness in lower limb	0.0066
	Pain and distension of anus	0.008
	Uterine bleeding	0.065
	Urinary retention	0.0015
	Hematuria	0.002
	Fever	0.0012
	Respiratory tract infection	0.0007
	Skin burn (1st and 2nd degree)	0.0012
	Nausea and vomiting	0.0155
	Dizziness and headache	0.0014
	Blurred vision	0.0073
Zhang CHJ et al., 2017 [4]	Lower abdominal pain	0.69
	Sciatic/buttock pain	0.57
	Skin burns	0.38
	Transient leg pain	0.34
	Vaginal bleeding after HIFU	0.15
Lozinski T et al., 2021 [19]	Abdominal pain	4.16
	Low-grade fever Flu-like symptomsmalaise, chills	2.43
	Hematuria	2.77
	Panic (claustrophobia)	0.33
Wang Y et al., 2018 [22]	**MRgHIFU**:	
	Abnormal vaginal discharge	0.046
	Lower abdominal pain	0.069
	**USgHIFU**	
	Abnormal vaginal discharge	0.05
	Lower abdominal pain	0.039
	Mild lower back pain	0.019

HIFU: high intensity focused ultrasound; MRgHIFU: MRI guided HIFU; USgHIFU: ultrasound guided HIFU.

### 3.8. Fibroid Volume Reduction

Resolution of symptoms and reduction in fibroid- and uterine volume are the indicators of treatment efficacy. Nevertheless, there is heterogeneity regarding the way this reduction was measured: some studies reported changes in the dominant or single fibroid and others documented all treated fibroids.

Most of the studies showed overall fibroid volume shrinkage ranging from 18% at three months [32] up to 84% at 36 months [3,17,18,19,37] (Table 6). The reduction depends on the initial fibroid volume and that there is a positive relationship between fibroid shrinkage and NPV ratio; the more the NPV, the higher the volume reduction [19]. Yu L, et al. [29] found in his meta-analysis higher reduction rates after USgHIFU (65.55%) than in MRgHIFU 36.44% for patients having <300 cm^3^ fibroid volume. He M, et al. [17] observed no significant difference in reduction rate between hypointense, isointense and hyperintense fibroids at one, three and six months (32.5% ± 24.0, 42.3 ± 32.2% and 52.5 ± 36.3%, respectively). Studies made with portable-HIFU showed 45.1 ± 25% volume reduction five months after therapy, because the treatment beam speed of portable-HIFU is similar to the speed obtained with MRgHIFU (1.30 mL/min ± 1.08 mL/min vs. 1.49 mL/min) [17].

In comparison with other non-interventional techniques, reduction in volume was observed after HIFU (34% ± 8%) than other techniques at 24 months follow-up (UAE 70% ± 11% and RFA (83% ± 8%) [34]. This difference with the aforementioned studies could be attributed to the fact that the initial fibroid volume was higher in the UAE and HIFU groups (UAE: 208.32 ± 112.41, HIFU 209.98 ± 77.83, RFA 84.35 ± 85.06).

**Table 6:** Myoma volume reduction after HIFU.

Author [Ref]	Myoma Localization	Myoma Volume before Intervention Mean (cm^3^)	Myoma Volume Reduction Immediately after Intervention (%)	Myoma Volume Reduction Up to 6 Months (cm^3^)	Myoma Volume Reduction at Final Follow-Up (cm^3^)
Jeng CJ et al., 2020 [3]	Posterior wall 34.7%	193.9 ± 458.0	NA	118.7 ± 240.0 at 3 months	40.2 ± 21.6 cm^3^ at 3 months
	Anterior wall 35.7%			NA	NA
	Fundal 8.7%			NA	NA
	Anterior and posterior wall 20.9%			NA	NA
Cheung VYT et al., 2019 [37]	Not categorized	127.0 (18.5–481.2)	79.8 % (6.6–271.7) at 1 month	46.9% (8.8–73.1) at 1 month	75.9% (33.7–99.3) at 12 months
He M et al., 2018 [17]	Anterior 127 cases	1.8–1220.1	NA	32.5 ± 24.0% at 1 month	NA
	Posterior 72 cases			52.5 ± 36.3%, at 6 months	
	Lateral 67 cases				
	Fundal 80 cases				
Lee JY et al., 2019 [18]	Anterior 19 cases Posterior 10 case Lateral 14 cases Fundus 10 cases	5.3 ± 1.5	NA	17.3 ± 30.0% at 1 month 33.3 ± 19.3% at 3 months (1st trial) 45.1 ± 25.5% at 5 months (2nd trial)	33.3 ± 19.3% at 3 months (1st trial) 45.1 ± 25.5% at 5 months (2nd trial)
Lozinski T et al., 2021 [19]		113.18 ± 1.96	NA	3 months: 27%	6 months: 39%

NA: not available.

### 3.9. Reintervention

Ten papers estimated reintervention rates after HIFU (Table 7), and report that MRgHIFU has higher reintervention rates than other ablation methods. In the meta-analysis from Yu L et al. [29] evaluating 11 studies for fibroids <300 cm^3^, the summarized reintervention rate was 8.1% (I^2^ = 8.1%; π^2^ = 0.0015; *p* < 0.01), with MRgHIFU presenting higher rates than USgHIFU (mean 13.4%; 95% CI: 5.4–21.4 vs. 5.2%; 95% CI: 2.0–8.4). In another meta-analysis, Wang Y et al. [22] reported that reintervention rate is higher in HIFU as compared to UAE (pooled OR: 11.99, 95% CI: 5.17–27.83, *p* < 0.01) and myomectomy (pooled OR: 4.05, 95% CI95% CI: 1.82–8.9). Likewise, Liu L et al. [8] reported that reintervention was significantly more frequent in USgHIFU as compared to UAE (pooled RR 0.25; 95% CI, 0.15–0.42, *p* < 0.001; I^2^ = 0%), attributed to the difference in myoma ablation mechanism between these techniques. Additionally, Jeng et al. CJ [28] reported in their meta-analysis there was a 7.1% greater risk of reintervention in MRgHIFU within 12 months than UAE in all four revised studies (Pooled OR = 6.843; 95% CI = 2.473 to 18.936, *p* < 0.001). Similarly, in the RCT study from Laughlin-Tommaso S, et al. [16] the reintervention rate within three years was higher after MRgHIFU than UAE (56.9% vs. 23.0%; HR, 2.81; 95% CI, 1.01–7.79) for patients with higher baseline anti-müllerian hormone level (AMH) than median (0.3 ng/mL). The reintervention rate at three years in women with low AMH levels was lower in both groups (22.1% MRgHIFU vs. 0.0% UAE).

**Figure 2:**
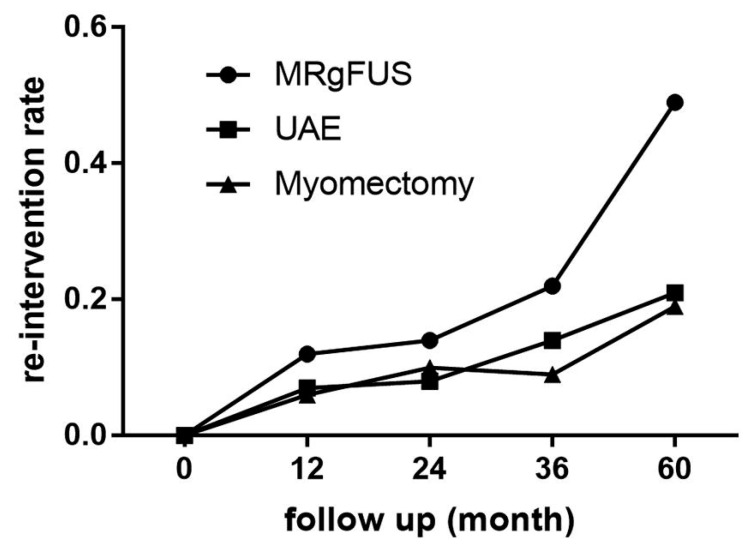
Reintervention rate after first non-surgical intervention of uterine fibroids. Pooled analysis of 42,103 patients. MRgHIFU: UAE: uterine artery embolization. Reprinted with permission from: Xu F et al. The comparison of myomectomy, UAE and MRgFUS in the treatment of uterine fibroids: a metaanalysis [33].

When comparing MRgHIFU with UAE and myomectomy, in the meta-analysis conducted by Xu F, et al. [33], up to 60 months of follow-up in all groups showed a steady incline of reintervention (Figure 2). This significant statistic difference is markedly seen after 24 months for each treatment, being higher for MRgHIFU group at the end of the follow-up period (49%). In the meta-analysis conducted by Sandberg EM, et al. [31] a reintervention in the following six months after therapy was necessary in 12.2% of cases after myomectomy, in 7% after hysteroscopic myomectomy, and in 14.4% after UAE.

Regarding the reasons for reintervention (Table 7), a recent cohort study with long follow-up period (70 ± 9.0 months) showed a 20.7% (79/381) overall reintervention rate after USgHIFU, which was due to the relapse of symptoms (50/79, 63.3%), psychological factors (14/79, 17.7%), infertility (3/79, 3.8%), suspected uterine sarcoma (2/79, 2.5%) and others (10/79, 12.7%) [23].

Furthermore, HIFU has been reported to be a good option for patients who have recurrent symptomatic uterine fibroids after myomectomy, because they will not need renewed surgical procedures. Liu X et al. [24] found out that after HIFU the symptom recurrence-time leading to a reintervention is longer as compared to secondary myomectomy (54 vs. 34 months; *p* = 0.024). The cumulative risk of reintervention at one and three years after myomectomy was higher than after HIFU (4.8% vs. 0%; 11.9% vs. 3.2%, respectively), which was attributed to surgical complications because of the first myomectomy. However, no significant difference was found between secondary myomectomy and HIFU at 5 and 8 years (15.5% vs. 9.5%; 17.9% vs. 13.7%, respectively) [24].

**Table 7:** Reintervention rates, reasons for reintervention and further procedure.

Author	HIFU Cases (*n*)	Re-Intervened (%)	Reintervention Interval (Months)	Reasons (%)	Further Procedure
Wang Y et al., 2020 [15]	245	24 (9.80%)	78	Symptoms recurred	Myomectomy (*n* = 8)
					Hysterectomy (*n* = 8)
					HIFU (*n* = 5)
					Hormone treatment (*n* = 3)
Li W et al., 2020 [23]	381	79 (20.73%)	33.7 ± 18.0	Symptomatic recurred, 63.3%	Myomectomy (*n* = 46)
				Psychological factors, 17.7%	Hysterectomy (*n* = 30)
				Fertility requirement, 3.8%	USgHIFU (*n* = 3)
				Suspected uterine sarcoma, 2.5%	
				Others, 10%	
He M et al., 2018 [17]	132	2 (1.51%)	6	Menstrual bleeding	Myomectomy
Cheung VYT et al., 2019 [14]	20	3 (12%)	10–12	Symptoms recurred	Myomectomy

HIFU: high intensity focused ultrasound.

### 3.10. Pregnancy Outcome

A total of 656 pregnancies were reported in six different studies [3,8,17,25,26,35] (Table 8). Wu G, et al. [25] compared retrospective pregnancy outcomes between one to eight years after USgHIFU (*n* = 248) and laparoscopic myomectomy (*n* = 253). From all 443 post intervention pregnancies, 68.4% occurred after USgHIFU group and 66.7% after myomectomy; 405 occurred spontaneously and 38 were after in vitro fertilization. They found that average times to pregnancy were statistically significantly higher after myomectomy (18.9 ± 7.3 months vs. 13.6 ± 9.5; *p* < 0.05). After USgHIFU spontaneous vaginal delivery was more frequent (51.1% vs. 36.4%) and caesarean section rate was lower (41.6% vs. 54.9%). Some pregnancy-related complications (Table 8) were lower after HIFU including preterm labor, placenta previa and placenta accreta [25,26,35], but after myomectomy, lower rates of preterm birth, fetal distress, fetal growth restriction and puerperal infection were observed. The rate of uterine rupture was the same in both groups (0.6%) [25].

He M et al. [17] evaluated the pregnancy rate in African women with pregnancy desire up to 6 months after HIFU and myomectomy. Out of 81 patients, only one was conceived after 3 months of completed HIFU for multiple fibroids and had a term vaginal delivery without any obstetrical complication.

Comparing to UAE, the meta-analysis of Liu L et al. [8] reported that HIFU has significantly higher pregnancy rates than UAE (18.0% to 25.0% vs. 0%; overall RR 0.06; 95% CI, 0.01–0.45; *p* = 0.006; I^2^ = 0%; 157 women). This difference could be due to selection bias, because older women, having larger size myomata, underwent UAE more frequently than HIFU.

In addition, Qu K, et al. [38] reported that ovarian reserve is probably not impaired by HIFU. They evaluated the AMH level of 67 patients prior to USgHIFU in accordance to age, <35 years, 36–40 years, >40 years. A significant difference in AMH level was observed in the group of patients with ages between 36 and 40 years, but no significant difference in AMH levels was observed in the other two groups (*p* > 0.05). Hence, the short follow-up period of this study does not allow to elucidate the impact of AMH-level changes on pregnancy rates of women between 36 and 40 years-old.

**Table 8:** Pregnancy outcomes after HIFU therapy.

Author	Approach (n)	Follow Up Duration	Time to Conceive (Months)	No. of Pregnancies	No. of Deliveries	SVD (n)	CS (n)	Complications
Wu G et al., 2020 [25]	USgHIFU: 219	1–8 years	USgHIFU: 13.6 ± 9.5	USgHIFU: 248	USgHIFU: 178	USgHIFU: 91	USgHIFU: 74	**Fetal distress**: USgHIFU: 2.8% LM 1.2% *p* = 0.468
	LM: 224		LM: 18.9 ± 7.3	**LM**: 253	LM: 173	LM: 63	LM: 95	**Fetal growth** **restriction**: USgHIFU: 2.2% LM:0.6%, *p* = 0.385
								**Large infant**: USgHIFU: 7.9% LM: 5.8%, *p* = 0.439
								**Placental abruption**: USgHIFU: 3.9%, LM: 4.6%, *p* = 0.74
								**Placenta increta**: USgHIFU: 1.1%, LM: 6.4%, *p* = 0.009
								**Placenta previa**: USgHIFU: 2.8%, LM: 8.7%, *p* = 0.018
								**Uterine rupture**: USgHIFU: 0.6%, LM: 0.6%, *p* = 1.000
Jeng CJ et al., 2020 [3]	546	3–38 months	NA	12	5	3	2	NA
Liu X et al., 2018 [26]	174	76 months	16 (1–66)	88	Not available	37	37	Placenta previa: 1
								Pregnancy induced hypertension: 1
								Fetal intrauterine growth retardation: 1
								Low birth weight: 2
								Malpresentation: 4
								Postpartum hemorrhage: 2
He M et al., 2018 [17]	1	6 months	3	1	1	1	0	NA
Liu L et al., 2020 [8]	157	6–61.9 months	NA	18	NA	NA	NA	NA
Anneveldt KJ et al., 2021 [35]	MRgHIFU: 114	6 months	MRgHIFU: 0–30	MRgHIFU: 124	MRgHIFU: 90	MRgHIFU: 71	MRgHIFU: 19	Placenta previa: MRgHIFU: 2/124 USgHIFU: 6/266
	USgHIFU: 325	1–8 years	USgHIFU: 1–66	USgHIFU: 336	USgHIFU: 248	USgHIFU: 64	USgHIFU: 184	Still births: MRgHIFU: 0 USgHIFU: 1

HIFU: high intensity focused ultrasound; MRgHIFU: MRI guided HIFU; USgHIFU: ultrasound guided HIFU; SVD: spontaneous vaginal delivery; CS: caesarean section.

Recently, Anneveldt KJ, et al. [35] retrospectively analyzed 21 studies on reproductive outcomes after MRgHIFU and USgHIFU. They found that the time to conceive is reported to be longer in USgHIFU groups (mean, 8 m vs. 16 m). From a total of 460 pregnancies, miscarriage was observed higher after MRgHIFU (0% to 50%) than after USgHIFU (0% to 15%) and live-birth rate was lower after MRgHIFU (73% vs. 91%).

Furthermore, the recent prospective study conducted by Liu X, et al. [26] including 174 women having desire of pregnancy, a total of 88 pregnancies (46.6%) occurred after USgHIFU within a median follow up period of 76 months. In total, 10% (9/88) of cases presented were miscarriages and 6% (5/88) chose elective termination. Caesarean section was elected by 50% of women, 11 additional caesarean sections were performed due to pregnancy-related complications and the rest successfully delivered vaginally.

### 3.11. Risk of Uterine Sarcoma

The incidence of sarcoma after HIFU has been reported by few research groups. Wang Q et al. [27] reported that from 15,713 patients who underwent HIFU, six cases (0.038%) were histologically confirmed but without dissemination signs at surgery. Interestingly, after data-imaging review, the NPV ratio of all these malignancies was high (86.8 ± 11.7%), indicating that sarcoma tissue could respond to HIFU. Furthermore, there was no statistical difference in overall survival rate of the six mentioned patients in comparison to 11 patients diagnosed with sarcoma who underwent surgery as first therapy during the same period (2008–2019). A slight lower incidence of sarcoma (1/3810; 0.26%) was reported by Li W et al. [23] within the follow-up period (58 m to 88 m) of patients who underwent USgHIFU. In this case, data-imaging review showed clues of sarcoma at pre-treatment MRI, including hyperintensity on T2WI and moderate enhancement on contrast MRI. The post-therapy MRI at three months showed fibroid regrowth and similar features, which was confirmed at surgery; the patient was free of disease five years later. Moreover, HIFU in combination with chemotherapeutic agents has been tested in a patient who presented a recurrent and difficult leiomyosarcoma [39]. Authors reported that patient’s survival time was prolonged because tumor growth was controlled. It was hypothesized that the acoustic cavitation, radiometric force, shear stress and acoustic stream produced by HIFU could make tumor cells more sensitive to chemotherapeutic agents, especially low vascularized cells in resting period.

## 4. Discussion

It is well known that the introduction of new technologies in medical practice usually takes a long time, from device development up to approval by regulatory agencies. Moreover, their acceptance and introduction in guidelines as alternative to standard therapies can also depend on its affordability, especially in low-income countries. In the case of uterine myomatosis, surgical treatments impose a large burden on women’s QoL and costs to health systems worldwide. Therefore, it is important to gain access to effective and safe minimally invasive approaches that increase the women´s chances to preserve their uterus and fertility and, at the same, to have good cosmetic results. In the last decade, HIFU has been introduced as a promising technology, able to reduce fibroid volume and symptoms, and it is expected to be introduced in European countries. Until now, most of the evidence has been published from Asiatic countries, in other languages than English, possibly influencing this opinion paper.

The working principle of HIFU therapy is to use ultrasonic waves, guided by MRI or ultrasound, in order to ablate the tissue through thermal and cavitation effects that result in destruction of parenchymal and vascular endothelial cells of tumor and, finally, in fibroid size shrinkage and symptomatic relief [40]. The effective volume reduction depends mainly on size and localization of fibroids and achieved NPV ratio; the higher NPV achieved (>70%), the more fibroid size reduction is to be expected [8,12]. An important factor that strongly attenuates ultrasonic waves is the subcutaneous abdominal fat layer, leading to lower NPV ratios [10]; in consequence, the pre-treatment assessment of patient is necessary to calculate the beam intensity. In terms of preventing surrounding organ damage and to avoid major complications, it is suggested that USgHIFU could be better than MRgHIFU, because patients do not require sedation and could give feedback to the physician about pain during procedure, allowing physicians to relocate the beam or to modulate its intensity [15].

In order to avoid damage to nearby organs and determining the beam intensity to ablate the fibroids, distance from skin to tumor, as well as localization, number, size and perfusion pattern of fibroids should be evaluated by means of ultrasound or MRI before commencing therapy. Post procedure imaging should be completed to evaluate the achieved NPV ratio of treated area [12]. MRI, with or without contrast, is considered the standard examination method in most of developed countries. Thus, MRI is quite expensive, time consuming, not available in all medical centers and its cost mostly not covered by insurance companies. As an alternative, contrast enhanced ultrasound could be used before procedure because of its lower cost, allowing real time analysis [30]. Moreover, USgHIFU is more cost effective than MRgHIFU and UAE, as it can be performed by a gynecologist skilled to perform pelvic ultrasound. Secondly, patients are not exposed to ionizing radiation like UAE. Lastly, MRI is not needed during the procedure, so a radiologist is not needed. Hence, it is in accordance with the lean management approach of health organizations to optimize resources [41].

Several studies showed lower frequency of major complications and treatable minor complications, in which skin redness, abdominal pain or discomfort and sciatic nerve paresthesia or simply leg pain are the most common. SAE occur in less than 0.5% of cases including second degree skin burn, lesions to bowel, ureter and bladder [29]. These complications occur when organs are in acoustic pathway or when the thermal effect induces inflammatory response and edema [21]. In order to avoid complications, we recommend an accurate and individual pre-assessment of patients including physical and imaging examination by means of contrast enhanced ultrasound or MRI, good communication with patients prior to the procedure, strict preparation for degreasing and degassing of the skin and checking skin and patient reactions regularly during procedure.

Outcomes of HIFU depend on different matters. According to myoma localization, it is observed that intramural fibroids [11] with volume under 300 cm^3^ [29] show higher volume reduction results due to less deep tissue and scared blood supply, as compared to transmural and submucosal uterine fibroids. Regarding signal intensity of fibroids, two retrospective studies reported contradictory results about fibroid shrinkage. He M, et al. [17] found no significant difference between hypointense, isointense and hyperintense fibroids, while Fan HJ, et al. [11] showed that hypointense fibroids exhibit higher reduction rates. This difference could be explained because the main population in He M et al. study was black women while Fan HJ, et al. included only Chinese women. Thus, other efficacy and safety studies conducted with Black, White and Afro-American women show similar results than those for Asiatic populations [4,6]. Therefore, ethnicity would not represent a problem in implementing this technique worldwide. However, high quality controlled trials should be conducted involving patients of all ethnicities to assert the efficacy of HIFU in high-risk of myoma development populations and to determine the relationship between ethnicity and tumor reduction after therapy.

Besides fibroid shrinkage, risk of recurrence, necessity of reintervention, pregnancy and sarcoma risk are concerns related to non-surgical therapies. In the longest follow-up study [15], 36 months was the median recurrent time reported after HIFU (range, 10 to 100 m) and 44 months after myomectomy (range 8 m to 96 m). In relation to reintervention, all reviewed reports show that this rate is higher in HIFU groups as compared to other approaches, especially statistically significant lower rates are reported after UAE [7]. Furthermore, USgHIFU has lower reintervention rates than MRgHIFU [29]. These differences could be attributed to the difference in myoma ablation mechanism between all techniques, as well as, to size, localization and FIGO type of myoma, signal intensity on T2 weighted image (T2WI), ablation temperature and higher levels of anti-müllerian hormone before therapy [2,7,12,16], which potentially correlates with the higher age of patients frequently included in the UAE studies. Additionally, in a retrospective study, HIFU showed to be more effective than secondary myomectomy for the treatment of recurrent symptomatic myomata, with a longer symptom free period, lower reintervention frequency and fewer side effects [24]. Multiple myomata in younger patients have more chances to reoccur after HIFU; some patients might also need multiple HIFU sessions.

Regarding pregnancy outcomes, studies show that conception can occur during the first three months in more than 67% of women seeking to be pregnant [4,17], with the average time to pregnancy shorter being after USgHIFU than myomectomy [25]. The rate of pregnancy-related complications do not differ from general population or from other myoma interventions, but the rate of uterine rupture was found to be lower compared with the rates reported for women with more than one caesarean section (<1% vs. 3.9%) [42]. A long latency period between HIFU and conception for safety reasons does not seem to be necessary to allow adequate postsurgical healing [43], which is usually advised after a myomectomy [44]. This evidence indicates that HIFU does not impair directly the fertility, pregnancy or delivery, which is especially beneficial for premenopausal women already struggling with lower pregnancy chances [35].

Patients and physicians should be aware of recurrence risks and strict necessity of follow up, including physical examination and imaging conducted to detect early recurrence and to prevent misdiagnosis in leiomyosarcoma. It is unknown at present whether leiomyosarcoma represents de novo growth or malignant transformation from benign uterine fibroids. This rare malignant tumor, less than 1 in 1000 of uterine wall diseases, is characterized by a rapid increase of uterine size, abnormal bleeding, pain and, sometimes, general symptoms related to metastases [45]. A very low incidence of sarcoma after HIFU (0.038% to 0.26%) has been reported from two studies within a follow-up period up to 88 months, without statistical difference in OS rate in comparison to myomectomy [23,27]. Due to this serious concern about leiomyosarcoma, it is important to obtain informed consent from patients, as there is a lack of histological confirmation before and after procedure [46].

In addition to tumor response, we consider that improvement in QoL plays a pivotal role in the patient´s decision-making process to consent for therapeutically approaches. We found that HIFU and myomectomy have similar effects on QoL life after 12 months [30]. Although, after three months, HIFU showed better results than myomectomy in terms of pain scores, sexual satisfaction and symptoms related to compression by fibroids [3], while UAE showed better results in terms of symptoms alleviation than USgHIFU [3]. Improvements of QoL, tolerability and effectiveness of HIFU have been also observed in solid abdominal tumors other than uterine fibroids, as reported by Oxford HIFU research center [46]. Based on available evidence, HIFU could be a new safe and effective option for worldwide patients suffering from uterine myomatosis.

## 5. Key Points to Consider in Clinical Practice

In accordance to the results of our analysis and to facilitate the clinical decision-making process regarding myoma treatment, HIFU could be considered as an alternative non-surgical treatment of uterine fibroids, according to an individualized benefit/risk ratio, irrespective of women ethnicity, and when the following aspects have been considered:

- Localization, size and benign radiological appearance of the uterine masses are accurately assessed prior to ablation. It is more difficult to ablate myomata with long distance from its ventral side to skin, in retroverted uterus, significant enhancement on T1WI, hyperintensity SI on T2WI (MRI), small size, transmural type and posterior location;
- Myoma perfusion (volume blood flow and velocity) is evaluated before procedure, e.g., by contrast enhanced ultrasound. The higher the perfusion, the higher the resistance towards ablation;
- Beam intensity and total treatment time is modulated according to myoma characteristics and subcutaneous abdominal fat thickness. Obese patients are at a higher risk of thermal damage in abdominal wall structures;
- An adequate acoustic window is assured before ablation. Bowel injuries can occur when the bowel is in the acoustic pathway or when fibroids are over treated;
- Patients presenting recurrent symptomatic uterine fibroids after myomectomy benefit from ablation. Most of them do not require further surgery;
- Patients are informed that discharged necrotic tissue will appear in the first cycle after therapy and resolve spontaneously within six to eight months;
- Patients are informed that fibroid volume shrinkage is achieved months after first ablation (mean 6 m);
- Patients are informed that reintervention is mostly required in younger women, when baseline anti-müllerian hormone level is >0.3 ng/mL or when fibroma volume is >300 cm^3^;
- Patients seeking to be pregnant are informed that average times to pregnancy are longer than after myomectomy, but pregnancy outcomes are similar and caesarean section as mode of delivery is not mandatory;
- Patients undergoing USgHIFU are informed that radiologist and sedation are not mandatory, therefore they give feedback to the physician about pain during procedure eventually to relocate the beam or to modulate its intensity;
- Patients are informed about potential AE, such as skin redness, abdominal pain or discomfort and sciatic nerve paresthesia or simply leg pain, skin burn, and in very seldom cases, nearby organs injury;
- Close follow-up of patient regarding the risk of development of a uterine sarcoma, as the uterine fibroids are not previously sampled for a histological diagnosis.

## 6. Conclusions

Several studies have been conducted evaluating the safety and efficacy of HIFU and its impact on women’s quality of life, but the quality of evidence relies mostly on retrospective cohort studies and meta-analysis (EBM Level IIa, IIb) conducted in Asiatic countries with a scarce representation of other populations. They report a low frequency of side effects, speedy recovery and high patient satisfaction; thus, treatment efficacy is similar to UAE, but better than surgical approaches. However, there are few reports differentiating the outcomes in non-Asiatic white populations, Afro-American and black women. We consider that ethnicity would not be a problem to implement this therapy in other countries, but the gap in the evidence should be evaluated in further trials involving different populations, to known whether ethnicity and other epigenetics factors could affect the outcomes of this new therapy. Additionally, it is necessary to prevent misdiagnosis of leiomyosarcoma, through strict imaging evaluation prior to therapy and close follow-up of patients.

## Figures and Tables

**Table 1:** Characteristics of the studies about HIFU included in this opinion paper.

Author [Ref]	Study Design	Level of Evidence	Country	Population No. of Patients/Ethnicity	Age of Participants (Years Old)
Jeng CJ et al., 2020 [3]	Retrospective, cross-sectional	II b	Taiwan	Myomatosis: 404, Adenomyosis: 149	40.7 ± 5.9
Zhang C et al., 2017 [4]	Retrospective non comparative	II b	South Africa	26 Black	34.4 ± 6.2
Liu Y et al., 2017 [6]	Prospective comparative	II b	China	HIFU: 99, LM: 67	>20
Suomi V et al., 2019 [10]	Retrospective, non comparative	II b	Finland	89; White: 80, Black: 6, Asian: 3	26–51
Fan HJ et al., 2018 [11]	Retrospective, non comparative	II b	China	207	18–55
Wang YJ et al., 2018 [12]	Retrospective, non comparative	II b	China	263	38.2 ± 5.6
Yin N et al., 2018 [13]	Prospective, non comparative	II b	China	892	39.1 ± 6.4
Cheung VYT et al., 2019 [14]	Prospective, non comparative	II b	Hong Kong	20	>40
Wang Y et al., 2020 [15]	Retrospective, comparative	II b	China	HIFU: 245, Uterus sparing surgery: 129	25–52; 23–53
Laughlin-Tommaso S et al., 2019 [16]	Randomized control trial	I b	USA	MRgHIFU: 43, UAE: 40	44.4
He M et al., 2017 [17]	Retrospective, comparative	II b	South Africa	176	35.3 ± 5.9
Lee JY et al., 2019 [18]	Prospective non comparative	II b	Korea	36	44.9 ± 28.6
Lozinski T et al., 2021 [19]	Prospective non comparative	II b	Poland	288	36.6 ± 5.3
Liu X et al., 2020 [20]	Retrospective, comparative	II b	China	96	39.15 ± 5.46
Liu Y et al., 2018 [21]	Retrospective, non comparative	II b	China	17402	37–45
Wang Y et al., 2018 [22]	Prospective, comparative	II b	China	MRgHIFU: 3 USgHIFU: 51	41.6 ± 5.5, 38.6 ± 7.0
Li W et al., 2020 [23]	Retrospective, non comparative	II b	China	381	40.7 ± 5.1
Liu X et al., 2020 [24]	Retrospective, comparative	II b	China	188	39.3 ± 5.9
Wu G et al., 2020 [25]	Retrospective, comparative	II b	China	HIFU: 320 LM:336	22–42
Liu X et al., 2018 [26]	Prospective non comparative	II b	China	81	31.1 ± 3.8
Wang Q et al., 2021 [27]	Retrospective non comparative	II b	China	15,759	40–47

HIFU = high intensity focused ultrasound; MRgHIFU: MRI guided HIFU; USgHIFU: ultrasound guided HIFU; LM: laparoscopic myomectomy, UAE: Uterine artery embolization.

**Table 2:** Outcomes after HIFU and other uterine sparing therapies reported by meta-analysis and systematic reviews.

Author [Ref]	Design/ (No. of Studies Included)	Objective	No. of Cases	Age	Results
Jeng CJ et al., 2020 [28]	Systematic review (4)	Comparison MRgHIFU with UAE for treatment of uterine myomata	HIFU: 207 UAE: 201	NA	**Symptom severity score**: I^2^ = 65.1%; Cochran Q = 5.7; *p* = 0.057 **QoL**: I^2^ = 0%; Cochran Q = 0.7; *p* = 0.697 **Reintervention rate**: I^2^ = 48.8%; Cochran Q: 5.9; *p* = 0.119 **AE**: I^2^ = 62.5%; Cochran Q = 5.3; *p* = 0.069
Yu L et al., 2021 [29]	Meta-analysis (48)	Efficacy and safety of MRgHIFU and USgHIFU in treatment of uterine fibroids with volume <300 cm^3^	MRgHIFU: 2179 USgHIFU: 4068	NA	**mean NPVR**: **MRgHIFU**: 58.92% (95% CI: 46.94–70.89), **USgHIFU**: 81.07% (95% CI: 77.61–84.53) **mean treatment time**: **MRgHIFU**: 178.19 min (95% CI: 140.24–216.15), **USgHIFU**: 96.9 min (95% CI: 82.20–111.60) **Skin thermal injury**: **USgHIFU**: 14.4% (95% CI: 7.3–21.5), **MRgHIFU**: 4.5% (95% CI: 2.5–6.4), **Sciatic nerve pain**: **MRgHIFU**: 8.9% (95% CI: 3.8–12.3), **USgHIFU**: 15.7% (95% CI: 8.2–23.3), **Abdominal pain**: **MRgHIFU**: 37.0% (95% CI: 21.9–52.2), **USgHIFU**: 31.2% (95% CI: 21.2–41.1), **Abnormal vaginal discharge**: **MRgHIFU**: 20.3% (95% CI: 9.2–31.3), **USgHIFU**: 11.3% (95% CI: 7.7–14.9) **Reduction rate of UF**: **USgHIFU**: 3 mo: 42.42% (95% CI: 30.66–54.17), 6 mo: 58.72% (95% CI: 52.26–65.17), 12 mo: 65.55% (95% CI: 49.54–81.56), **MRgHIFU**: 3 mo: 34.79% (95% CI: 30.76–38.83), 6 mo: 37.79% (95% CI: 26.71–49.23), 12 mo: 36.44% (95% CI: 24.49–48.38). **One year reintervention rate**: **MRgHIFU**: 13.4% (95% CI: 5.4–21.4), **USgHIFU**: 5.2% (95% CI: 2.0–8.4)
Wang Y et al., 2021 [7]	Meta-analysis (18)	Compare effectiveness and safety of HIFU with myomectomy and hysterectomy		33.60–46.54	**Rate of reintervention**: **HIFU vs. UAE** (pooled OR: 11.99, 95% CI: 5.17–27.83, *p* < 0.01), **HIFU vs. MYO** (pooled OR: 4.05, 95% CI: 1.82–8.99, *p* < 0.01), **Incidence of abnormal pregnancy**: **HIFU vs. UAE**: (OR: 1.20, 95% CI: 0.42–3.40, *p* = 0.73), **HIFU vs. MYO**: (pooled OR: 0.82, 95% CI: 0.46–1.46, *p* = 0.50). **Change of serum sex hormones**: **HIFU vs. UAE**: FSH (MD: −0.20, 95% CI: −0.91–0.51, *p* = 0.58), LH (MD: 0.10, 95% CI: −0.55–0.75, *p* = 0.76), and E2 (MD: −1.00, 95% CI: −7.42–5.42, *p* = 0.76) **Days of hospital stay**: **HIFU vs. MYO**: (pooled MD: −4.70, 95% CI: −7.46–1.94, *p* < 0.01), **HIFU vs. HYS**: (MD: −6.90, 95% CI: −7.24–6.56, *p* < 0.01).
Liu L et al., 2020 [8]	Meta-analysis (7)	To compare the clinical effects of uterine artery embolization (UAE) with those of high-intensity focused ultrasound (HIFU) ablation for the treatment of symptomatic uterine fibroids	HIFU: 227 UAE: 4365	HIFU: 36.1–44.0 UAE: 41.2–46.0	**Change in UFS score 12 months**: MD or RR (95% CI), **UAE vs. HIFU**:19.54 (15.21–23.87), *p* < 0.001, I^2^: 0% **Changes in QoL score at 12 months**: MD or RR (95% CI), **UAE vs. HIFU**: 15.72 (8.30–23.13), *p* < 0.001, I^2^: 73%
					**Adverse events**: MD or RR (95% CI), **UAE vs. HIFU**: 3.42 (0.07–158.04), *p* = 0.53, I^2^ = 86% **Pregnancy rate**: MD or RR (95% CI), **UAE vs. HIFU**: 0.06 (0.01–0.45), *p* = 0.006, I^2^: 0% reintervention rates: MD or RR (95% CI), **UAE vs. HIFU**: 0.25 (0.15–0.42), *p* < 0.001, I^2^: 52%
Torkzaban M et al., 2020 [30]	Systematic review (17)	Clinical application and safety of contrasted enhanced ultrasound (CEUS)			CEUS provide detailed data about fibroid volume, vascularization during and post UAE. Intraprocedural CEUS during HIFU faster volume shrinkage with less needed energy and early detection of residual tissue.
Sandberg EM et al., 2018 [31]	Meta-analysis (85)	To compare uterine sparing treatment options for fibroids in terms of reintervention risk and quality of life	Myomectomy: 17,789 UAE: 5114 Artery ligation: 8244 Laparoscopic 50 RFA: 652 MRg/USg)–HIFU: 1548 Laparo-ablation: 20 Hysteroscopy: 1741 RFA: 120	29.3–47.9	**Quality of life at 12 months**: HIFU: 24.5 (95% CI: 90.8 to 18.1), I^2^: 96.9%. **Reintervention risks**: 60 months LM 12.2%, UAE 14.4%, HIFU 59.3%
Verpalena IM et al., 2019 [32]	Meta-analysis (16)	Reevaluation of effectiveness of MRgHIFU for uterine fibroids by excluding studies with restrictive protocols that no longer used	1323	NA	**NPV**: Overall (I^2^ = 99.38%, *p* = 0.000), 95% CI: 68.1% (59.9–76.0%) **tSSS**: Overall (I^2^ = 94.46%, *p* < 0.001), 95% CI: 43.001 (34.300, 51.701), Overall (I^2^ = 97.87%, *p* < 0.001) 95% CI: 49.265(39.989, 58.541), Overall (I^2^ = 75.35%, *p* = 0.0001) 95% CI: 59.875 (53.673, 66.078) **QoL**: Overall (I^2^ = 98.33%, *p* < 0.001) 31.444 (−16.275, 79,162), Overall (I^2^ = 99.76%, *p* < 0.001) 31.458 (−5.585, 68.501) **Fibroid shrinkage**: Overall (I^2^ = 82.1%, *p* < 0.001), 95% CI: 33.162 (27.865, 38.460), Overall (I^2^ = 96.8%, *p* < 0.001) 95% CI: 36.620 (28.942, 44.298), Overall (I^2^ = 0%, *p* = 0.0986) 95% CI: 37.742 (32.696, 42.789) **AE**: Overall (I^2^ = 79.04%, *p* = 0.000), 95% CI: 0.087 (0.057, 0.132)
Xu F et al., 2021 [33]	Meta-analysis (31)	Comparison of reintervention rates of myomectomy, UAE and MRgHIFU in different follow up times	42103	NA	**Re-intervention rates**: 12 months re-intervention Myomectomy: 0.06 (95% CI, 0.01–0.11; I^2^ = 95.1%; *p* = 0.000). UAE: 0.07 (95% CI, 0.06–0.09; I^2^ = 14.2%; *p* = 0.324) MRgFUS 0.12 (95% CI, 0.04–0.20; I^2^ = 89.1%; *p* = 0.000) **36-month re-intervention**: MRgFUS: 0.22 (95% CI, 0.11–0.32; I^2^ = 86.3%; *p* = 0.002) UAE 0.22 (95% CI, 0.11–0.32; I^2^ = 86.3%; *p* = 0.002) myomectomy 0.09 (95% CI, 0.05–0.13; I^2^ = 0.0%; *p* = 0.508) **60-month reintervention**: MRgFUS 0.49 (95% CI, 0.21–0.77; I^2^ = 96.5%; *p* = 0.000) UAE: 0.21 (95% CI, 0.17–0.25; I^2^ = 84.1%; *p* = 0.000) myomectomy: 0.19 (95% CI, 0.15–0.24; I^2^ = 53.7%; *p* = 0.071)
Taheri M et al., 2019 [34]	Systematic review (81)	Examines the changes in uterine and fibroid volume in UAE, HIFU and RFA	NA	32.4–52	Pooled mean fibroid volume reduction ± SD: **UAE**: 3 mo: 44% ± 9%; 6 mo: 54% ± 10%; 9 mo: 61%; 12 mo: 66% ± 10%; 24 mo: 70% ± 11% **RFA**: 3 mo: 55% ± 9%; 6 mo: 70% ± 5%; 9 mo: 78%; 12 mo: 75% ± 15%; 24 mo: 83% ± 8%; 36 mo: 84%10%, HIFU: 3 mo: 21% ± 6%; 6 mo: 32% ± 11%; 12 mo: 28% ± 16%; 24 mo: 34% ± 8%; 36 mo: 32%
Anneveldt KJ et al., 2021 [35]	Systematic review (21)	Reproductive outcomes in Mg HIFU and USgHIFU	276	NA	47% pregnancy rate after 76 month f-up 90% live birth rate median time to conceive 16 months (1–66 months) 72–80% delivered by caesarian section

HIFU = high intensity focused ultrasound; MRgHIFU: MRI guided HIFU; USgHIFU: ultrasound guided HIFU; LM: laparoscopic myomectomy; UF: Uterine fibroid; UAE: Uterine artery embolization; AE: adverse event; CEUS: contrast enhanced ultrasound; NPV: non-perfused volume; NPVR: non-perfused volume ratio; RFA: radio frequency ablation; NA: not available. The Role of Ethnicity and Technical Parameters in HIFU Outcomes.

**Table 4:** Frequency of major adverse events after HIFU therapy reported in clinical studies.

Author [Ref]	Major Adverse Events	Frequency (%)
Jeng CJ et al., 2020 [3]	Urinary retention	0.001
	Acute renal failure	0.0003
	Bowel perforation	0.0001
	Abdominal hernia	0.00009
	Thrombocytopenia	0.00009
	Leg and buttock pain	0.00019
	Fever	0.0004
Chen J et al., 2018 [37]	Second degree skin burn	0.0022
Liu Y et al., 2018 [28]	Skin burns	0.149
	Leg pain	0.057
	Urinary retention	0.040
	Vaginal bleeding	0.034
	Hyperpyrexia	0.028
	Renal failure	0.023
	Acute cystitis	0.017
	Bowel injury	0.017
	Deep vein thrombosis	0.115
	Hydronephrosis	0.0057
	Thrombocytopenia	0.00005
	Intrauterine infection	0.011
Liu X et al., 2020 [20]	Pelvic adhesions after HIFU	0.43
	No pelvic adhesions after HIFU	0.54

## Data Availability

Not applicable.

## Abbreviations

HIFU: high intensity focused ultrasound; MRgHIFU: MRI guided HIFU; USgHIFU: ultrasound guided HIFU; LM: laparoscopic myomectomy; UAE: uterine artery embolization; NPVR: Non-perfused volume ratio; NPV: non-perfused volume; FVS: fibroid shrinkage volume; CEUS: contrast enhanced ultrasound; EEF: the energy efficiency factor; QoL, health-related quality of life; UFS-QOL: Uterine Fibroid Symptom and Quality of Life instrument; tSSS: transformed symptom severity scale; SVD: spontaneous vaginal delivery; CS: caesarean section.
